# Infections, inflammation, and risk of neuropsychiatric disorders: the neglected role of “co-infection”

**DOI:** 10.1016/j.heliyon.2020.e05645

**Published:** 2020-12-08

**Authors:** Amir Abdoli, Ali Taghipour, Majid Pirestani, Mirza Ali Mofazzal Jahromi, Abazar Roustazadeh, Hamed Mir, Hoda Mirzaian Ardakani, Azra Kenarkoohi, Shahab Falahi, Mahdi Karimi

**Affiliations:** a Department of Parasitology and Mycology, School of Medicine, Jahrom University of Medical Sciences, Jahrom, Iran; b Zoonoses Research Center, Jahrom University of Medical Sciences, Jahrom, Iran; c Department of Parasitology, Faculty of Medical Sciences, Tarbiat Modares University, Tehran, Iran; d Department of Advanced Medical Sciences & Technologies, School of Medicine, Jahrom University of Medical Sciences, Jahrom, Iran; e Department of Laboratory Sciences, School of Medicine, Jahrom University of Medical Sciences, Jahrom, Iran; f Research Center for Noncommunicable Diseases, School of Medicine, Jahrom University of Medical Sciences, Jahrom, Iran; g Department of Clinical Biochemistry, School of Medicine, Jahrom University of Medical Sciences, Jahrom, Iran; h Department of Microbiology, Faculty of Medicine, Ilam University of Medical Sciences, Ilam, Iran; i Zoonotic Diseases Research Center, Ilam University of Medical Sciences, Ilam, Iran; j Cellular and Molecular Research Center, Iran University of Medical Sciences, Tehran, Iran; k Department of Medical Nanotechnology, Faculty of Advanced Technologies in Medicine, Iran University of Medical Sciences, Tehran, Iran; l Advances Nanobiotechnology and Nanomedicine Research Group (ANNRG), Iran University of Medical Sciences, Tehran, Iran

**Keywords:** Infection, Inflammation, Neuropsychiatric disorders, Co-infection, Immunology, Microbiology, Infectious disease, Psychiatry

## Abstract

Neuropsychiatric disorders (NPDs) have multiple etiological factors, mainly genetic background, environmental conditions and immunological factors. The host immune responses play a pivotal role in various physiological and pathophysiological process. In NPDs, inflammatory immune responses have shown to be involved in diseases severity and treatment outcome. Inflammatory cytokines and chemokines are involved in various neurobiological pathways, such as GABAergic signaling and neurotransmitter synthesis. Infectious agents are among the major amplifier of inflammatory reactions, hence, have an indirect role in the pathogenesis of NPDs. As such, some infections directly affect the central nervous system (CNS) and alter the genes that involved in neurobiological pathways and NPDs. Interestingly, the most of infectious agents that involved in NPDs (e.g., *Toxoplasma gondii*, cytomegalovirus and herpes simplex virus) is latent (asymptomatic) and co-or-multiple infection of them are common. Nonetheless, the role of co-or-multiple infection in the pathogenesis of NPDs has not deeply investigated. Evidences indicate that co-or-multiple infection synergically augment the level of inflammatory reactions and have more severe outcomes than single infection. Hence, it is plausible that co-or-multiple infections can increase the risk and/or pathogenesis of NPDs. Further understanding about the role of co-or-multiple infections can offer new insights about the etiology, treatment and prevention of NPDs. Likewise, therapy based on anti-infective and anti-inflammatory agents could be a promising therapeutic option as an adjuvant for treatment of NPDs.

## 1 Introduction

Neuropsychiatric disorders (NPDs) are among the most important morbidity and mortality worldwide [1,2]. According to the estimation, the global burden of mental illness accounts for 13.0% of disability-adjusted life-years (DALYs) and 32·4% of years lived with disability (YLDs) [1]. Several factors, including environmental conditions, genetic background, immune dysregulation and some infectious agents are known to be involved in the etiopathogenesis of NPDs [3, 4, 5, 6]. In recent years, different investigations have shown the roles of inflammation in the etiopathogenesis of NPDs and anti-inflammatory agents as a therapeutic target of NPDs [7]. On the other hand, association of different NPDs with some infectious agents (Table 1), especially maternal infections have been demonstrated in various studies [8, 9]. Beyond the direct effects on the CNS, infections can augment inflammatory pathways, and consequently may have indirect roles in the etiopathogenesis of NPDs [10, 11, 12]. As well, the most of infectious agents that involved in NPDs, such as *Toxoplasma gondii* (*T. gondii*), cytomegalovirus (CMV), herpes simplex virus (HSV) and Epstein Barr virus (EBV) is asymptomatic (latent) and co-or-multiple infection of them are common [13, 14, 15, 16, 17]. Although association of single infection with NPDs have been investigated in many studies [18, 19, 20, 21], little is known about the association of co-or-multiple infections and NPDs. Because co-or-multiple infections have more adverse outcome than single infection [22, 23, 24, 25, 26, 27, 28, 29, 30], the major question of this article is: May co-or-multiple infections enhance the risk of NPDs? And, what is the possible association of co-or-multiple infections with NPDs?

**Table 1:** A snapshot on microbiology of the major infectious agents that involved in NPDs.

Infectious agent	Microbiology, transmission, and prevalence	Major symptoms
*T. gondii*	►*Toxoplasma gondii* is a protozoan parasite that infects most species of warm-blooded animals, including humans, and causes the disease toxoplasmosis. ►Domestic cats are only definitive host and warm-blooded animals and human are intermediated hosts. ►Infection usually occurs by eating undercooked contaminated meat, drinking contaminated water and foods, exposure from infected cat feces, mother-to-child transmission during pregnancy, receiving an infected organ transplant or infected blood via transfusion (https://www.cdc.gov/parasites/toxoplasmosis/gen_info/faqs.html). ►According to estimation, more than one of the third of the human population have a history of *T. gondii* infection [74].	►Most infected individuals are not aware of it because they have no symptoms at all. ►Some people who have toxoplasmosis may feel as if they have the “flu” with swollen lymph glands or muscle aches and pains that may last for a month or more. ►Severe toxoplasmosis, causing damage to the brain, eyes, or other organs and is more likely in individuals who have immunocompromising conditions, such as HIV/AIDS, organ recipient individuals, and patients with malignancies. (https://www.cdc.gov/parasites/toxoplasmosis/gen_info/faqs.html).
CMV	►CMV is a common virus for people of all ages with a seroprevalence ranging from 45 to 100% [75]. ►CMV infected individuals may pass the virus in body fluids, such as urine, saliva, tears, blood, semen, and breast milk. Hence, the infection can spread through direct contact with the infected body fluids, sexual contact, breast milk, transplanted organs and blood transfusions (https://www.cdc.gov/cmv/overview.html).	►Most immunocompetent individuals do not have symptoms, however, a flu-like symptoms may be detected in the first time of infection. ►Severe symptoms and fatal disease may develop in immunocompromised patients, and newborn babies with congenital CMV infection (https://www.cdc.gov/cmv/overview.html).
HSV	►The HSV is categorized into 2 types: HSV-1 and HSV-2. Both HSV-1 and HSV-2 infections are lifelong. ►HSV-1 is mainly transmitted by oral-to-oral contact to cause oral herpes (known as “cold sores”), but can also cause genital herpes. An estimated 3.7 billion people under age 50 (67%) have HSV-1 infection globally. ►HSV-1 is mainly transmitted by oral-to-oral contact to cause oral herpes infection, via contact with the HSV-1 virus in sores, saliva, and surfaces in or around the mouth. However, HSV-1 can also be transmitted to the genital area through oral-genital contact to cause genital herpes. ►HSV-2 is a sexually transmitted infection that causes genital herpes. An estimated 491 million people aged 15–49 (13%) worldwide have HSV-2 infection. ►HSV-2 is mainly transmitted during sex, through contact with genital surfaces, skin, sores or fluids of someone infected with the virus. HSV-2 can be transmitted from skin in the genital or anal area that looks normal and is often transmitted in the absence of symptoms (https://www.who.int/news-room/fact-sheets/detail/herpes-simplex-virus).	►Most oral and genital herpes infections are asymptomatic. ►Symptoms of herpes include painful blisters or ulcers at the site of infection. ► HSV-2 is amongst the most common infections in people living with HIV, occurring in 60–90% of HIV-infected persons. HSV-2 infection increases the risk of acquiring a new HIV infection by approximately three-fold. Moreover, people with both HIV and HSV-2 infection are more likely to spread HIV to others (https://www.who.int/news-room/fact-sheets/detail/herpes-simplex-virus).
Rubella	►Rubella, also known as German measles, is a contagious viral infection that occurs most often in children and young adults. ► Rubella infection in pregnant women may cause fetal death or congenital defects known as congenital rubella syndrome (CRS). ►There is no specific treatment for rubella but the disease is preventable by vaccination. ►The rubella virus is transmitted by airborne droplets when infected people sneeze or cough. Humans are the only known host (https://www.who.int/news-room/fact-sheets/detail/rubella).	►Rubella virus infection usually causes a mild fever and rash in children and adults, infection during pregnancy, especially during the first trimester, can result in miscarriage, fetal death, stillbirth, or infants with congenital malformations, known as congenital rubella syndrome (CRS). ►In children, the disease is usually mild, with symptoms including a rash, low fever (<39 °C), nausea and mild conjunctivitis. The rash, which occurs in 50–80% of cases and lasts 1–3 days. Swollen lymph glands behind the ears and in the neck are the most characteristic clinical feature. ►Infected adults, more commonly women, may develop arthritis and painful joints that usually last from 3–10 days. Children with CRS can suffer hearing impairments, eye and heart defects and other lifelong disabilities, including autism, diabetes mellitus and thyroid dysfunction. ►The highest risk of CRS is in countries where women of childbearing age do not have immunity to the disease (either through vaccination or from having had rubella) (https://www.who.int/news-room/fact-sheets/detail/rubella).
EBV	►EBV, also known as human herpesvirus 4, is a member of the herpes virus family. It is one of the most common human viruses worldwide. ►EBV spreads most commonly through bodily fluids, primarily saliva (https://www.cdc.gov/epstein-barr/about-ebv.html).	►EBV can cause infectious mononucleosis, also called mono, and other illnesses. ►Many people become infected with EBV in childhood. EBV infections in children usually do not cause symptoms. People who get symptoms from EBV infection, usually teenagers or adults, get better in two to four weeks. However, some people may feel fatigued for several weeks or even months. ►The virus becomes latent after infection in some cases, the virus may reactivate. This does not always cause symptoms, but people with weakened immune systems are more likely to develop symptoms if EBV reactivates (https://www.cdc.gov/epstein-barr/about-ebv.html).
Influenza	►Flu is a contagious respiratory illness caused by influenza viruses that infect the nose, throat, and sometimes the lungs. ►Flu viruses spread mainly by tiny droplets made when people with flu cough, sneeze or talk (https://www.cdc.gov/flu/about/keyfacts.htm).	►Influenza (flu) can cause mild to severe illness, and at times can lead to death. Flu usually comes on suddenly. ►Children are most likely to get sick from flu and that people 65 and older are least likely to get sick. ►Symptoms may be included fever, cough, sore throat, runny or stuffy nose, muscle or body aches, headaches, fatigue. Some people may have vomiting and diarrhea, though this is more common in children than adults (https://www.cdc.gov/flu/about/keyfacts.htm).
VZV	►Varicella-zoster virus (VZV) causes chickenpox and herpes zoster (shingles). ►Once the illness resolves, the virus remains latent in the dorsal root ganglia. VZV can reactive later in a person's life and cause a painful, maculopapular rash called herpes zoster.	►Chickenpox follows initial exposure to the virus and is typically a relatively mild, self-limited childhood illness with a characteristic exanthem, but can become disseminated in immunocompromised children. (https://emedicine.medscape.com/article/231927-overview) ►Postherpetic neuralgia (PHN) is the most common complication of herpes zoster. PHN is pain that persists in the area where the rash once was for more than 90 days after rash onset. PHN can last for weeks or months, and occasionally, for years. ►A person's risk of having PHN after herpes zoster increases with age. Older adults are more likely to have longer lasting, more severe pain. (https://www.cdc.gov/shingles/hcp/clinical-overview.html)
BDV	►BDV first described as a fatal neurologic disease of horses and sheep. Human infections have been described by serological and molecular methods [76].	►Human infection has been putative link to mental disorders, but the impact of BDV on mental-health still remains controversial [77].
*Chlamydia pneumoniae*	► *C. pneumoniae* causes respiratory tract infections, such as pneumonia. ►People spread *C. pneumoniae* by coughing or sneezing. Other people then breathe in the bacteria. People can also get sick if they touch something with droplets from a sick person on it and then touch their mouth or nose. (https://www.cdc.gov/pneumonia/atypical/cpneumoniae/about/index.html)	►In general, *C. pneumoniae* infection is a mild illness that most commonly causes an upper respiratory tract infection. These upper respiratory tract infections can include a sore throat or an ear or sinus infection. ►*C. pneumoniae* can also cause lower respiratory tract infections like bronchitis and lung infections like pneumonia. (https://www.cdc.gov/pneumonia/atypical/cpneumoniae/about/index.html)

## 2 Theory/hypothesis

Several indirect links proposed that co-or-multiple infections may be more involved in the etiopathogenesis of NPDs than single infection: ***1***) Some infections are associated with NPDs; ***2***) infections are associated with inflammation; ***3***) inflammation is associated with NPDs; ***4***) co-or-multiple infections enhanced a higher level of inflammatory biomarkers than single infections; ***5***) co-or-multiple infections have more severe outcome than single infection; Hence ***6***) co-or-multiple infections may have more influence in the etiopathogenesis of NPDs than single infection.

## 3 Evidences of the hypothesis

### 3.1 Inflammation and NPDs

In recent years, psychoneuroimmunology is a hot topic issue in NPDs and different studies have been focused on the role of immune disturbances in the etiology of NPDs [5, 31]. As reviewed elsewhere, inflammatory mediators are able to interact with multiple biological pathways related to NPDs, such as neuroendocrine activity, synaptic plasticity, neurocircuits as well as neurotransmitters and monoamine metabolism [31, 32]. For instance, neuroinflammation activate the kynurenine pathway that can modulate the N-methyl-D-aspartate (NMDA) receptor and diminish serotonin production, which consequently is involved in several NPDs, such as depressive disorders [8]. Inflammatory cytokines IL-6 and TNF-α increase blood-brain barrier (BBB) permeability, and blocking of them decreases stress-induced BBB opening [33, 34]. The result of a recent meta-analysis [12] demonstrated a significant increase in the levels of IL-17, IL-23, IL-6, TNF-α, soluble IL-2 receptor (sIL-2R), and IL-1 receptor antagonist (IL-1RA) in acutely ill patients with major depressive disorder (MDD), schizophrenia, and bipolar mania compared with controls (*P* < 0.01). Indeed, a significant increase in the levels of IL-1β and sIL-2R was detected in patients with chronic schizophrenia and bipolar disorder [12]. Recent researches have shown that macrophage migration inhibitory factor (MIF) plays a protective role in the development of MDD by upregulating the PI3k/Akt/mTOR pathway and production of inflammatory cytokines IL-1β and TNFα [35, 36, 37]. On the other hand, *in-vitro*, *in-vivo*, and ex-vivo preclinical data, as well as data from Alzheimer' s disease patients have shown that MIF is increased during the course of the disease and that therapeutic targeting of MIF could beneficial effects on the Alzheimer' s disease course [38].

**Figure 1:**
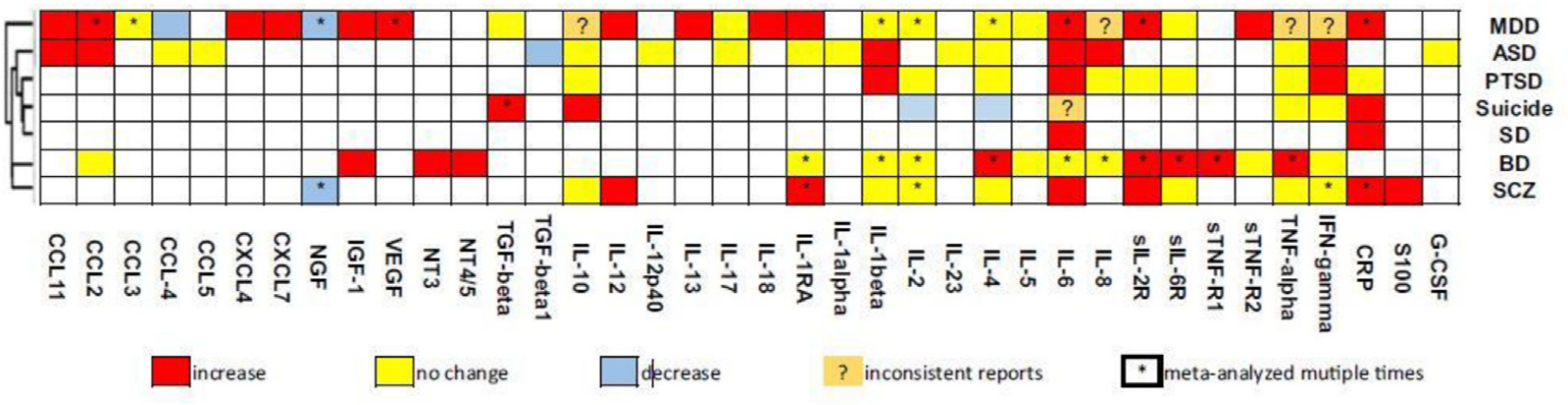
Alterations of 38 inflammatory mediators in patients with different NPDs. schizophrenia (SCZ), bipolar disorder (BD), autism spectrum disorder (ASD), major depression disorder (MDD), post-trauma stress disorder (PTSD), sleeping disorder (SD), obsessive–compulsive disorder (OCD) and suicide. Reproduced from reference [39] *(Open access article under a Creative Commons Attribution 4.0 International License).*

In an excellent article, Yuan et al. [39] performed an umbrella review of the meta-analyses regarding alterations of 38 inflammation-related factors in major NPDs. This study summarized the changes of different cytokines, chemokines, and growth factors in NPDs (Figure 1). Functionally, cytokines and chemokine are divided into proinflammatory and anti-inflammatory subsets. Abnormal levels of both pro-and anti-inflammatory cytokines and chemokine were detected in several NPDs (Figure 1) [39, 40, 41].

### 3.2 Autoimmunity and NPDs

The association between autoimmune disorders with NPDs is another evidence that link between inflammation and NPDs [42, 43, 44]. So, higher co-morbidity of some autoimmune diseases and NPDs have been reported from the epidemiological investigations [44, 45, 46]. In a recent meta-analysis, Siegmann et al. [46] showed that patients with autoimmune thyroiditis had significantly higher scores on depression (OR = 3.56) and anxiety disorders (OR = 2.32). Depression and anxiety disorders are increased in patients with multiple sclerosis (MS) as well [47]. In a cohort study among 5084 MS patients, the OR of depression and anxiety disorders was 1.4 and 1.23 in the prediagnostic period and the post-diagnostic period, respectively [48]. Rossi et al. [48] found that depression and anxiety disorders significantly increase inflammatory cytokines TNF-α, IL1-β, and IL-2 in the CSF samples of MS patients in the relapsing-remitting stage of the disease [49]. T_H_17 cells which has a highly inflammatory properties plays a critical pathogenic role in several autoimmune diseases [31]. Among the psoriasis patients, anti-IL-17A therapy resulted in remission of depression in about 40% of the patients with severe depression [50]. Depressive disorders are not only common in patients with diabetes [51, 52], but also increase risk of mortality among these patients (HR = 1.46) [53]. Psychiatric co-morbidity is also common in patients with other autoimmune disorders, such as inflammatory bowel disease (IBD) [54, 55], Systemic lupus erythematosus (SLE) [56] and psoriasis [57].

### 3.3 Anti-inflammatory treatment for NPDs

Anti-inflammatory agents can be used as an adjuvant in combination with anti-psychotic drugs [58]. The results of different meta-analysis revealed a diminished level of depressive symptoms after anti-inflammatory treatment [59]. Indeed, nonsteroidal anti-inflammatory drugs significantly improved the positive and negative symptoms of schizophrenia patients [60]. In contrast, Quereda and colleagues [61] showed that treatment with Efavirenz and α-interferon in patients with hepatitis C virus (HCV) and HIV co-infection were partially led to mood disorders. On the other hand, it has been suggested that an imbalanced production of cytokines may be involved in the pathogenesis and maintenance of NPDs. Taken together, cumulative evidences suggest the beneficial role of anti-inflammatory treatment as an adjuvant in treatment of NPDs.

### 3.4 Antipsychotic therapy modulates inflammatory biomarkers of NPDs

Another evidence that is shown the association of inflammation and NPDs is the effects of antipsychotic therapy on levels of inflammatory biomarkers. While clinical and experimental evidences reveal that the major antipsychotic agents, including lithium, haloperidol, valproate acid, perazine, clomipramine, fluoxetine and the selective serotonin reuptake inhibitors (SSRIs) led to modulation of inflammatory biomarkers [58, 62, 63]. For instance, the SSRIs decreased peripheral levels of IL-1β, IL-6, TNF-α and IL-10 [64]. *In vitro* studies also revealed that inflammatory cytokines IL-1β, TNF-α, and Nuclear factor kappa B (NF-κB) production were significantly impeded after exposure to haloperidol [65, 66]. A recent meta-analysis demonstrated that clomipramine and fluoxetine decrease inflammatory cytokines IL-6, TNF-α, and IFN-γ, whilst venlafaxine and mirtazapine augment their levels [63].

### 3.5 Association of latent infections with NPDs

**Figure 2:**
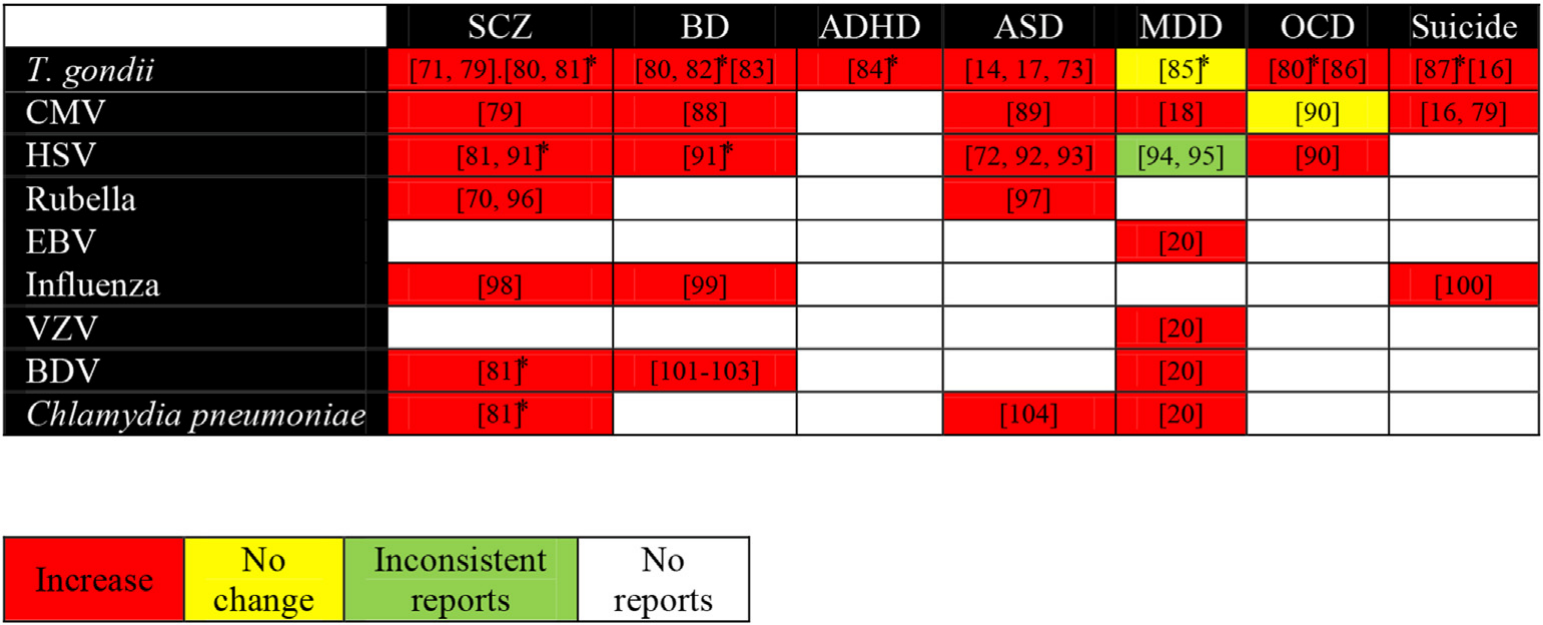
Association of single infections with NPDs. The related references are inserted in each box. ∗ Meta-analysis. [79], [80], [81], [82], [83], [84], [85], [86], [87], [88], [89], [90], [91], [92], [93], [94], [95], [96], [97], [98], [99], [100], [101].

**Figure 3:**
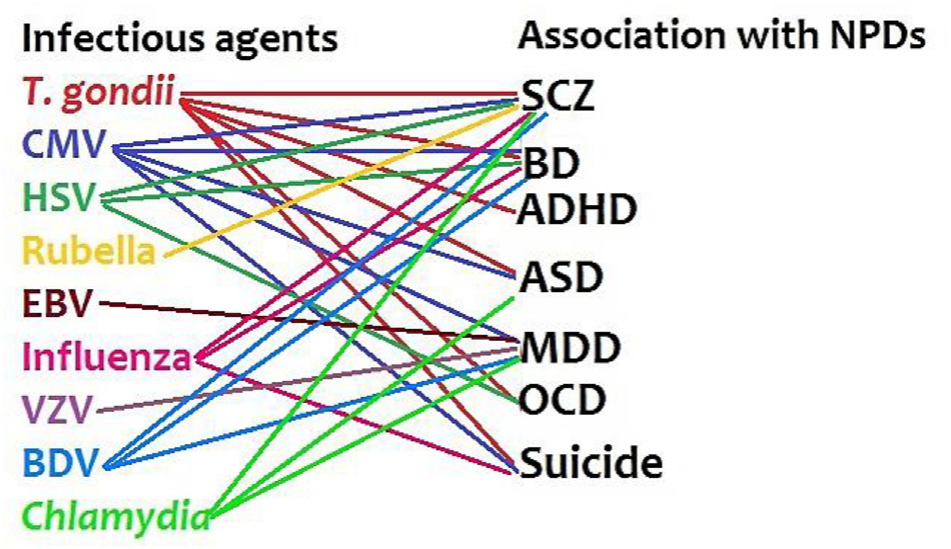
Association of infectious agents with NPDs (based on Figure 2).

Inflammation is induced by multiple stimulating factors, including infectious agents [67]. Hence, infections act as an inflammation amplifier which consequently involves in the etiopathogenesis of NPDs. Till now, a number of researches demonstrated a positive correlation of different NPDs with several infectious agents (Table 1), including *T. gondii*, CMV, HSV, EBV, rubella, measles, influenza, Borna disease virus (BDV), varicella zoster virus (VZV) [13, 14, 19, 68], and *Chlamydia* [18, 20] infection (Figures 2 and 3). Beyond the direct effects of infections on the central nervous system (CNS), infections affect the immune system that leads to product inflammatory mediators, such as immune cells and cytokines. These mediators can pass through the blood-brain barrier (BBB) and generate neuroinflammation [8]. As such, maternal infections in pregnant women can increase risk of NPDs in the offspring in later life [14, 17, 69, 70, 71, 72]. On the other hand, depression increased the risk of infections among women with coronary artery bypass grafting compared to non-depressed women [73]. Despite the positive correlations between infections and NPDs, limited information is available about the role of co-or multiple-infections in the etiopathogenesis of NPDs.

### 3.6 Evidences for synergetic role of co-or-multiple infections on outcome of the diseases

Quite a few studies have demonstrated that co-or-multiple infections synergically enhance severity of the diseases. For example, viral co-infection has several virological and immunological consequences, such as enhanced virus replication and persistence, altered disease intensity and altered immunological responses [24]. In the “Spanish flu” pandemic, 95% of the mortality was attributed to co-infection with bacterial pneumonia as well [26]. Co-infection of *Streptococcus pneumoniae* with influenza virus promotes inflammatory responses with a strong IL-17A response that led to enhanced *S. pneumoniae* disease intensity in the nasopharynx of infected animals [103]. *In vitro* study in the human monocytic cell lines revealed that co-exposure of influenza virus with *Staphylococcus aureus* toxins enhanced pro-inflammatory cytokines, such as TNF-α, IL-1β, and IL-6 [23]. HIV and hepatitis C virus co-infection promote hepatocellular injury that is linked to elevation of certain inflammatory cytokines [104]. In the HIV-infected patients, co-infection with human herpesvirus 8 (HHV-8) was associated with a persistent inflammation and immune activation [25]. Previous studies showed that maternal infection with ToRCH (toxoplasmosis, rubella, CMV and HSV) co-infection was associated with increased risk of abortion in pregnant women than their single infection [27]. As such, some studies indicated that co-infections synergically enhance the level of inflammatory mediators. In this regard, Souza et al. [30] reported that chronic infection with *T. gondii* exacerbates secondary polymicrobial sepsis in an experimental mouse model and in a human survey. The results revealed that chronic *T. gondii* infection suppresses anti-inflammatory T helper (T_H_)-2 cells and simultaneously intensifies local and systemic inflammatory T_H_1 cells and their inflammatory cytokines, such as IFN-γ and nitric oxide (NO) [30]. These phenomena were resulted in reduced diastolic and systolic blood pressures after induction of sepsis and led to a severe outcome than uninfected *T. gondii* mice with sepsis [30]. A clinical study was also performed by the same group of the researchers [30] regarding the correlation of *T. gondii* seropositivity with inflammatory biomarkers in patients with sepsis. They found that the sepsis severity was positively correlated with increased IFN-γ levels in *T. gondii* seropositive patients compare with *T. gondii* seronegative septic patients [30]. Hence, accumulating evidence reveals that co-or-multiple infections have more severe outcomes than single infection [22].

### 3.7 Possible role of co-or-multiple infections in increased risk of NPDs

**Figure 4:**
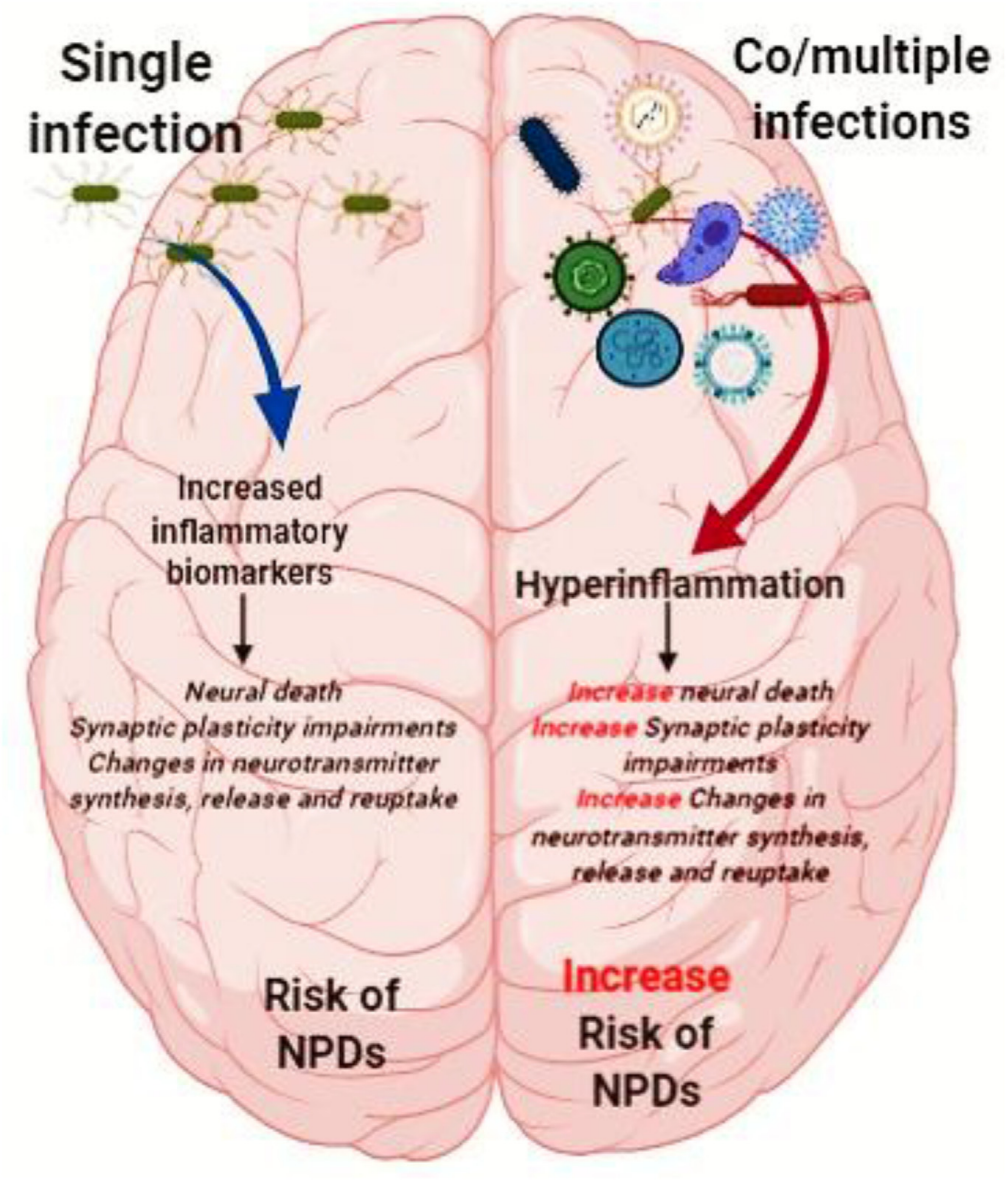
A hypothetical scheme on the possible role of single infection or co-or-multiple infections in the pathogenesis of NPDs. (The figure designed by BioRender online software).

In a large-scale study among Danish individuals with various psychiatric disorders, Burgdorf and colleagues [78] found a significant association between *T. gondii* and schizophrenia, and between CMV and attempting or committing suicide, neurotic, stress-related and somatoform disorders, and mood disorders. Nevertheless, *T. gondii* and CMV co-infection did not influence the overall findings [78]. Although, the levels of inflammatory markers were not reported in this study [78]. Nicolson et al [102] found that prevalence of either single infection or co-infection of *Mycoplasma* ssp., *Chlamydia pneumoniae*, and Human Herpes Virus-6 were higher in patients with ASD than the control group. Some studies have shown that patients with HCV and HIV co-infection may be at higher risk for depressive disorders than single infection [105, 106, 107]. Aibibula and colleagues [105] demonstrated that HIV patients with depressive symptoms had 1.32 times higher risk of HIV viremia. As such, in HIV-HCV co-infected patients, occurrence of depressive symptoms were a risk factor for persistent HIV viremia [105]. To our knowledge, there are not any immunological analyses regarding the influences of co-or-multiple infections in the etiopathogenesis of NPDs until now. Hence, it seems that co-or-multiple infections is a neglected topic in the area of NPDs (Figure 4).

## 4 Conclusion and future directions

As mentioned, various studies have shown the role of single infections in the etiopathogenesis of NPDs, but, the role of co-infections have not deeply investigated. Torrey and Yolken [6] mentioned that the role of genetic background in the etiology of schizophrenia may have been overestimated and an increased attention to gene-environmental interactions can accelerate research development on this disease. Furthermore, interaction of infectious agents with microbiota composition can produce a better clinical picture of NPDs [6]. The idea of infectious cause NPDs may open new opportunities for treatment of NPDs with some antibiotics, antiviral or antiprotozoal agents. As well, investigations on the role co-or-multiple infections can provide new insides into the pivotal role of infectious in the etiopathogenesis of NPDs rather than other etiological factors (e.g., genetic background). Further understanding about the influences of co-or-multiple on NPDs can provide new insights about the etiology, treatment, and prevention of NPDs.

## Declarations

### Author contribution statement

All authors listed have significantly contributed to the development and the writing of this article.

### Funding statement

This research did not receive any specific grant from funding agencies in the public, commercial, or not-for-profit sectors.

### Data availability statement

No data was used for the research described in the article.

### Declaration of interests statement

The authors declare no conflict of interest.

### Additional information

No additional information is available for this paper.

## References

[1] Vigo D., Thornicroft G., Atun R. (2016). Estimating the true global burden of mental illness. Lancet Psych..

[2] Walker E.R., McGee R.E., Druss B.G. (2015). Mortality in mental disorders and global disease burden implications: a systematic review and meta-analysis mental disorder Mortality Mental disorder mortality. JAMA Psych..

[3] Burmeister M., McInnis M.G., Zöllner S. (2008). Psychiatric genetics: progress amid controversy. Nat. Rev. Genet..

[4] van Os J., Kenis G., Rutten B.P. (2010). The environment and schizophrenia. Nature.

[5] Khandaker G.M., Cousins L., Deakin J., Lennox B.R., Yolken R., Jones P.B. (2015). Inflammation and immunity in schizophrenia: implications for pathophysiology and treatment. Lancet Psych..

[6] Torrey E.F., Yolken R.H. (2019). Schizophrenia as a pseudogenetic disease: a call for more gene-environmental studies. Psychiatr. Res..

[7] Pape K., Tamouza R., Leboyer M., Zipp F. (2019). Immunoneuropsychiatry — novel perspectives on brain disorders. Nat. Rev. Neurol..

[8] Labrie V., Brundin L. (2018). Harbingers of mental disease—infections associated with an increased risk for neuropsychiatric illness in children. JAMA Psych..

[9] Köhler-Forsberg O., Petersen L., Gasse C., Mortensen P.B., Dalsgaard S., Yolken R.H. (2019). A nationwide study in Denmark of the association between treated infections and the subsequent risk of treated mental disorders in children and adolescents. JAMA Psych..

[10] Fernandes B.S., Steiner J., Molendijk M.L., Dodd S., Nardin P., Gonçalves C.-A. (2016). C-reactive protein concentrations across the mood spectrum in bipolar disorder: a systematic review and meta-analysis. Lancet Psych..

[11] Fraguas D., Díaz-Caneja C.M., Ayora M., Hernández-Álvarez F., Rodríguez-Quiroga A., Recio S. (2018). Oxidative stress and inflammation in first-episode psychosis: a systematic review and meta-analysis. Schizophr. Bull..

[12] Goldsmith D., Rapaport M., Miller B. (2016). A meta-analysis of blood cytokine network alterations in psychiatric patients: comparisons between schizophrenia, bipolar disorder and depression. Mol. Psychiatr..

[13] Abdoli A., Dalimi A. (2014). Are there any relationships between latent Toxoplasma gondii infection, testosterone elevation, and risk of autism spectrum disorder?. Front. Behav. Neurosci..

[14] Abdoli A., Dalimi A., Arbabi M., Ghaffarifar F. (2014). Neuropsychiatric manifestations of latent toxoplasmosis on mothers and their offspring. J. Matern. Fetal Neonatal Med..

[15] Coryell W., Wilcox H., Evans S.J., Pandey G.N., Jones-Brando L., Dickerson F. (2020). Latent infection, inflammatory markers and suicide attempt history in depressive disorders. J. Affect. Disord..

[16] Flegr J., Horacek J. (2019). Negative effects of latent toxoplasmosis on mental health. Front. Psychiatr..

[17] Ford B.N., Yolken R.H., Aupperle R.L., Teague T.K., Irwin M.R., Paulus M.P. (2019). Association of early-life stress with cytomegalovirus infection in adults with major depressive disorder. JAMA Psych..

[18] Arias I., Sorlozano A., Villegas E., de Dios Luna J., McKenney K., Cervilla J. (2012). Infectious agents associated with schizophrenia: a meta-analysis. Schizophr. Res..

[19] Wang X., Zhang L., Lei Y., Liu X., Zhou X., Liu Y. (2014). Meta-analysis of infectious agents and depression. Sci. Rep..

[20] Khandaker G., Zimbron J., Lewis G., Jones P. (2013). Prenatal maternal infection, neurodevelopment and adult schizophrenia: a systematic review of population-based studies. Psychol. Med..

[21] Orlovska S., Vestergaard C.H., Bech B.H., Nordentoft M., Vestergaard M., Benros M.E. (2017). Association of streptococcal throat infection with mental disorders: testing key aspects of the PANDAS hypothesis in a nationwide study. JAMA Psych..

[22] Hunter P. (2018). Co-infection: when whole can be greater than the sum: the complex reaction to co-infection of different pathogens can generate variable symptoms. EMBO Rep..

[23] Jeannoel M., Casalegno J.-S., Ottmann M., Badiou C., Dumitrescu O., Lina B. (2018). Synergistic effects of influenza and *Staphylococcus aureus* toxins on inflammation activation and cytotoxicity in human monocytic cell lines. Toxins.

[24] Kumar N., Sharma S., Barua S., Tripathi B.N., Rouse B.T. (2018). Virological and immunological outcomes of coinfections. Clin. Microbiol. Rev..

[25] Masiá M., Robledano C., de la Tabla V.O., Antequera P., Lumbreras B., Hernández I. (2014). Coinfection with human herpesvirus 8 is associated with persistent inflammation and immune activation in virologically suppressed HIV-infected patients. PloS One.

[26] Morens D.M., Taubenberger J.K., Fauci A.S. (2008). Predominant role of bacterial pneumonia as a cause of death in pandemic influenza: implications for pandemic influenza preparedness. J. Infect. Dis..

[27] Rasti S., Ghasemi F.S., Abdoli A., Piroozmand A., Mousavi S.G.A., Fakhrie-Kashan Z. (2016). ToRCH “co-infections” are associated with increased risk of abortion in pregnant women. Congenital. Anom..

[28] Rinaldo C.R., Richter B.S., Black P.H., Callery R., Chess L., Hirsch M.S. (1978). Replication of herpes simplex virus and cytomegalovirus in human leukocytes. J. Immunol..

[29] Shmagel K.V., Saidakova E.V., Shmagel N.G., Korolevskaya L.B., Chereshnev V.A., Robinson J. (2016). Systemic inflammation and liver damage in HIV/hepatitis C virus coinfection. HIV Med..

[30] Souza M.C., Fonseca D.M., Kanashiro A., Benevides L., Medina T.S., Dias M.S. (2017). Chronic *Toxoplasma gondii* infection exacerbates secondary polymicrobial sepsis. Front. Cell Infect. Microbiol..

[31] Beurel E., Toups M., Nemeroff C.B. (2020). The bidirectional relationship of depression and inflammation: double trouble. Neuron.

[32] Haroon E., Raison C.L., Miller A.H. (2012). Psychoneuroimmunology meets neuropsychopharmacology: translational implications of the impact of inflammation on behavior. Neuropsychopharmacology.

[33] Cheng Y., Desse S., Martinez A., Worthen R.J., Jope R.S., Beurel E. (2018). TNFα disrupts blood brain barrier integrity to maintain prolonged depressive-like behavior in mice. Brain Behav. Immun..

[34] Menard C., Pfau M.L., Hodes G.E., Kana V., Wang V.X., Bouchard S. (2017). Social stress induces neurovascular pathology promoting depression. Nat. Neurosci..

[35] Petralia M.C., Fagone P., Basile M.S., Lenzo V., Quattropani M., Bendtzen K. (2019). Pathogenic contribution of the Macrophage migration inhibitory factor family to major depressive disorder and emerging tailored therapeutic approaches. J. Affect. Disord..

[36] Günther S., Fagone P., Jalce G., Atanasov A.G., Guignabert C., Nicoletti F. (2019). Role of MIF and D-DT in immune-inflammatory, autoimmune, and chronic respiratory diseases: from pathogenic factors to therapeutic targets. Drug Discov. Today.

[37] Oliveira C.S., de Bock C.E., Molloy T.J., Sadeqzadeh E., Geng X.Y., Hersey P. (2014). Macrophage migration inhibitory factor engages PI3K/Akt signalling and is a prognostic factor in metastatic melanoma. BMC Canc..

[38] Petralia M.C., Battaglia G., Bruno V., Pennisi M., Mangano K., Lombardo S.D. (2020). The role of macrophage migration inhibitory factor in alzheimer′ s disease: conventionally pathogenetic or unconventionally protective?. Molecules.

[39] Yuan N., Chen Y., Xia Y., Dai J., Liu C. (2019). Inflammation-related biomarkers in major psychiatric disorders: a cross-disorder assessment of reproducibility and specificity in 43 meta-analyses. Transl. Psychiatry.

[40] Petralia M.C., Mazzon E., Fagone P., Basile M.S., Lenzo V., Quattropani M.C. (2020). The cytokine network in the pathogenesis of major depressive disorder. Close to translation?. Autoimmun. Rev..

[41] Stuart M.J., Baune B.T. (2014). Chemokines and chemokine receptors in mood disorders, schizophrenia, and cognitive impairment: a systematic review of biomarker studies. Neurosci. Biobehav. Rev..

[42] Bergink V., Gibney S.M., Drexhage H.A. (2014). Autoimmunity, inflammation, and psychosis: a search for peripheral markers. Biol. Psychiatr..

[43] Mané-Damas M., Hoffmann C., Zong S., Tan A., Molenaar P.C., Losen M. (2019). Autoimmunity in psychotic disorders. Where we stand, challenges and opportunities. Autoimmun. Rev..

[44] Petralia M.C., Mazzon E., Fagone P., Basile M.S., Lenzo V., Quattropani M.C. (2020). The cytokine network in the pathogenesis of major depressive disorder. Close to translation?. Autoimmun. Rev..

[45] Benros M.E., Eaton W.W., Mortensen P.B. (2014). The epidemiologic evidence linking autoimmune diseases and psychosis. Biol. Psychiatr..

[46] Siegmann E.-M., Müller H.H.O., Luecke C., Philipsen A., Kornhuber J., Grömer T.W. (2018). Association of depression and anxiety disorders with autoimmune thyroiditis: a systematic review and meta-analysis. JAMA Psych..

[47] Boeschoten R.E., Braamse A.M.J., Beekman A.T.F., Cuijpers P., van Oppen P., Dekker J. (2017). Prevalence of depression and anxiety in Multiple Sclerosis: a systematic review and meta-analysis. J. Neurol. Sci..

[48] Hoang H., Laursen B., Stenager E.N., Stenager E. (2016). Psychiatric co-morbidity in multiple sclerosis: the risk of depression and anxiety before and after MS diagnosis. Multiple Sclero. J..

[49] Rossi S., Studer V., Motta C., Polidoro S., Perugini J., Macchiarulo G. (2017). Neuroinflammation drives anxiety and depression in relapsing-remitting multiple sclerosis. Neurology.

[50] Griffiths C.E.M., Fava M., Miller A.H., Russell J., Ball S.G., Xu W. (2017). Impact of Ixekizumab treatment on depressive symptoms and systemic inflammation in patients with moderate-to-severe psoriasis: an integrated analysis of three phase 3 clinical studies. Psychother. Psychosom..

[51] Dybdal D., Tolstrup J.S., Sildorf S.M., Boisen K.A., Svensson J., Skovgaard A.M. (2018). Increasing risk of psychiatric morbidity after childhood onset type 1 diabetes: a population-based cohort study. Diabetologia.

[52] Gilsanz P., Karter A.J., Beeri M.S., Quesenberry C.P., Whitmer R.A. (2018). The bidirectional association between depression and severe hypoglycemic and hyperglycemic events in type 1 diabetes. Diabetes Care.

[53] van Dooren F.E.P., Nefs G., Schram M.T., Verhey F.R.J., Denollet J., Pouwer F. (2013). Depression and risk of mortality in people with diabetes mellitus: a systematic review and meta-analysis. PloS One.

[54] Frolkis A.D., Vallerand I.A., Shaheen A.-A., Lowerison M.W., Swain M.G., Barnabe C. (2019). Depression increases the risk of inflammatory bowel disease, which may be mitigated by the use of antidepressants in the treatment of depression. Gut.

[55] Moulton C.D., Pavlidis P., Norton C., Norton S., Pariante C., Hayee B. (2019). Depressive symptoms in inflammatory bowel disease: an extraintestinal manifestation of inflammation?. Clin. Exp. Immunol..

[56] Roberts A.L., Kubzansky L.D., Malspeis S., Feldman C.H., Costenbader K.H. (2018). Association of depression with risk of incident systemic lupus erythematosus in women assessed across 2 decades. JAMA Psych..

[57] Fleming P., Bai J.W., Pratt M., Sibbald C., Lynde C., Gulliver W.P. (2017). The prevalence of anxiety in patients with psoriasis: a systematic review of observational studies and clinical trials. J. Eur. Acad. Dermatol. Venereol..

[58] Sommer I.E., van Westrhenen R., Begemann M.J.H., de Witte L.D., Leucht S., Kahn R.S. (2013). Efficacy of anti-inflammatory agents to improve symptoms in patients with schizophrenia: an update. Schizophr. Bull..

[59] Köhler O., Benros M.E., Nordentoft M., Farkouh M.E., Iyengar R.L., Mors O. (2014). Effect of anti-inflammatory treatment on depression, depressive symptoms, and adverse effects: a systematic review and meta-analysis of randomized clinical trials. JAMA Psych..

[60] Sommer I.E., de Witte L., Begemann M., Kahn R.S. (2012). Nonsteroidal anti-inflammatory drugs in schizophrenia: ready for practice or a good start? A meta-analysis. J. Clin. Psychiatr..

[61] Quereda C., Corral I., Moreno A., Pérez-Elías M.J., Casado J.L., Dronda F. (2008). Effect of treatment with efavirenz on neuropsychiatric adverse events of interferon in HIV/HCV-coinfected patients. J. Acquir. Immune Defic. Syndr..

[62] Balõtšev R., Haring L., Koido K., Leping V., Kriisa K., Zilmer M. (2019). Antipsychotic treatment is associated with inflammatory and metabolic biomarkers alterations among first-episode psychosis patients: a 7-month follow-up study. Early Interven. Psych..

[63] Baumeister D., Ciufolini S., Mondelli V. (2016). Effects of psychotropic drugs on inflammation: consequence or mediator of therapeutic effects in psychiatric treatment?. Psychopharmacology.

[64] Wang L., Wang R., Liu L., Qiao D., Baldwin D.S., Hou R. (2019). Effects of SSRIs on peripheral inflammatory markers in patients with major depressive disorder: a systematic review and meta-analysis. Brain Behav. Immun..

[65] Moots R., Al-Saffar Z., Hutchinson D., Golding S., Young S., Bacon P. (1999). Old drug, new tricks: haloperidol inhibits secretion of proinflammatory cytokines. Ann. Rheum. Dis..

[66] Yamamoto S., Ohta N., Matsumoto A., Horiguchi Y., Koide M., Fujino Y. (2016). Haloperidol suppresses NF-kappaB to inhibit lipopolysaccharide-induced pro-inflammatory response in RAW 264 cells. Med. Sci. Mon..

[67] Johnson K.V.-A., Foster K.R. (2018). Why does the microbiome affect behaviour?. Nat. Rev. Microbiol..

[68] Tucker J.D., Bertke A.S. (2019). Assessment of cognitive impairment in HSV-1 positive schizophrenia and bipolar patients: systematic review and meta-analysis. Schizophr. Res..

[69] Brown A.S., Cohen P., Harkavy-Friedman J., Babulas V., Malaspina D., Gorman J.M. (2001). Prenatal rubella, premorbid abnormalities, and adult schizophrenia. Biol. Psychiatr..

[70] Brown A.S., Schaefer C.A., Quesenberry C.P., Liu L., Babulas V.P., Susser E.S. (2005). Maternal exposure to toxoplasmosis and risk of schizophrenia in adult offspring. Am. J. Psychiatr..

[71] Mahic M., Mjaaland S., Bøvelstad H.M., Gunnes N., Susser E., Bresnahan M. (2017). Maternal immunoreactivity to herpes simplex virus 2 and risk of autism spectrum disorder in male offspring. mSphere.

[72] Spann M.N., Sourander A., Surcel H.-M., Hinkka-Yli-Salomäki S., Brown A.S. (2017). Prenatal toxoplasmosis antibody and childhood autism. Autism Res..

[73] Doering L.V., Martínez-Maza O., Vredevoe D.L., Cowan M.J. (2008). Relation of depression, natural killer cell function, and infections after coronary artery bypass in women. Eur. J. Cardiovasc. Nurs..

[74] Tenter A.M., Heckeroth A.R., Weiss L.M. (2000). *Toxoplasma gondii*: from animals to humans. Int. J. Parasitol..

[75] Cannon M.J., Schmid D.S., Hyde T.B. (2010). Review of cytomegalovirus seroprevalence and demographic characteristics associated with infection. Rev. Med. Virol..

[76] Carbone K.M. (2001). Borna disease virus and human disease. Clin. Microbiol. Rev..

[77] Mazaheri-Tehrani E., Maghsoudi N., Shams J., Soori H., Atashi H., Motamedi F. (2014). Borna disease virus (BDV) infection in psychiatric patients and healthy controls in Iran. Virol. J..

[78] Burgdorf K.S., Trabjerg B.B., Pedersen M.G., Nissen J., Banasik K., Pedersen O.B. (2019). Large-scale study of *Toxoplasma* and Cytomegalovirus shows an association between infection and serious psychiatric disorders. Brain Behav. Immun..

[79] Sutterland A.L., Fond G., Kuin A., Koeter M.W.J., Lutter R., van Gool T. (2015). Beyond the association. *Toxoplasma gondii* in schizophrenia, bipolar disorder, and addiction: systematic review and meta-analysis. Acta Psychiatr. Scand..

[80] Arias I., Sorlozano A., Villegas E., Luna JdD., McKenney K., Cervilla J. (2012). Infectious agents associated with schizophrenia: a meta-analysis. Schizophr. Res..

[81] de Barros J.L.V.M., Barbosa I.G., Salem H., Rocha N.P., Kummer A., Okusaga O.O. (2017). Is there any association between *Toxoplasma gondii* infection and bipolar disorder? A systematic review and meta-analysis. J. Affect. Disord..

[82] Snijders G.J., van Mierlo H.C., Boks M.P., Begemann M.J., Sutterland A.L., Litjens M. (2019). The association between antibodies to neurotropic pathogens and bipolar disorder. Transl. Psychiatry.

[83] Nayeri T., Sarvi S., Moosazadeh M., Hosseininejad Z., Amouei A., Daryani A. (2020). *Toxoplasma gondii* infection and risk of attention-deficit hyperactivity disorder: a systematic review and meta-analysis. Pathog. Glob. Health.

[84] Chegeni T.N., Sharif M., Sarvi S., Moosazadeh M., Montazeri M., Aghayan S.A. (2019). Is there any association between *Toxoplasma gondii* infection and depression? A systematic review and meta-analysis. PloS One.

[85] Flegr J., Horáček J. (2017). *Toxoplasma*-infected subjects report an Obsessive-Compulsive Disorder diagnosis more often and score higher in Obsessive-Compulsive Inventory. Eur. Psychiatr..

[86] Sutterland A.L., Kuin A., Kuiper B., van Gool T., Leboyer M., Fond G. (2019). Driving us mad: the association of *Toxoplasma gondii* with suicide attempts and traffic accidents – a systematic review and meta-analysis. Psychol. Med..

[87] Frye M.A., Coombes B.J., McElroy S.L., Jones-Brando L., Bond D.J., Veldic M. (2019). Association of cytomegalovirus and *Toxoplasma gondii* antibody titers with bipolar disorder. JAMA Psych..

[88] Slawinski B.L., Talge N., Ingersoll B., Smith A., Glazier A., Kerver J. (2018). Maternal cytomegalovirus sero-positivity and autism symptoms in children. Am. J. Reprod. Immunol..

[89] Khanna S., Ravi V., Shenoy P.K., Chandramuki A., Channabasavanna S.M. (1997). Cerebrospinal fluid viral antibodies in obsessive compulsive disorder in an indian population. Biol. Psychiatr..

[90] Zappulo E., Riccio M.P., Binda S., Pellegrinelli L., Pregliasco F., Buonomo A.R. (2018). Prevalence of HSV1/2 congenital infection assessed through genome detection on dried blood spot in individuals with autism spectrum disorders. Vivo.

[91] Mora M., Quintero L., Cardenas R., Suarez-Roca H., Zavala M., Montiel N. (2009). Association between HSV-2 infection and serum anti-rat brain antibodies in patients with autism. Invest. Clin..

[92] Gale S.D., Berrett A.N., Erickson L.D., Brown B.L., Hedges D.W. (2018). Association between virus exposure and depression in US adults. Psychiatr. Res..

[93] Simanek A.M., Cheng C., Yolken R., Uddin M., Galea S., Aiello A.E. (2014). Herpesviruses, inflammatory markers and incident depression in a longitudinal study of Detroit residents. Psychoneuroendocrinology.

[94] Brown A.S., Cohen P., Greenwald S., Susser E. (2000). Nonaffective psychosis after prenatal exposure to rubella. Am. J. Psychiatr..

[95] Hutton J. (2016). Does rubella cause autism: a 2015 reappraisal?. Front. Hum. Neurosci..

[96] Brown A.S., Begg M.D., Gravenstein S., Schaefer C.A., Wyatt R.J., Bresnahan M. (2004). Serologic evidence of prenatal influenza in the etiology of schizophrenia. Arch. Gen. Psychiatr..

[97] Parboosing R., Bao Y., Shen L., Schaefer C.A., Brown A.S. (2013). Gestational influenza and bipolar disorder in adult offspring. JAMA Psych..

[98] Okusaga O., Yolken R.H., Langenberg P., Lapidus M., Arling T.A., Dickerson F.B. (2011). Association of seropositivity for influenza and coronaviruses with history of mood disorders and suicide attempts. J. Affect. Disord..

[99] Fu Z.F., Amsterdam J.D., Kao M., Sharikar V., Koprowski H., Dietzschold B. (1993). Detection of borna disease virus-reactive antibodies from patients with affective disorders by western immunoblot technique. J. Affect. Disord..

[100] Bode L., Dürrwald R., Rantam F., Ferszt R., Ludwig H. (1996). First isolates of infectious human Borna disease virus from patients with mood disorders. Mol. Psychiatr..

[101] Amsterdam J.D., Winokur A., Dyson W., Herzog S., Gonzalez F., Rott R. (1985). Borna disease virus: a possible etiologic factor in human affective disorders?. Arch. Gen. Psychiatr..

[102] Nicolson G.L., Gan R., Nicolson N.L., Haier J. (2007). Evidence for *Mycoplasma* ssp., *Chlamydia pneunomiae*, and human herpes virus-6 coinfections in the blood of patients with autistic spectrum disorders. J. Neurosci. Res..

[103] Ambigapathy G., Schmit T., Mathur R.K., Nookala S., Bahri S., Pirofski L-a (2019). Double-edged role of interleukin 17A in Streptococcus pneumoniae pathogenesis during influenza virus coinfection. J. Infect. Dis..

[104] Shmagel K., Saidakova E., Shmagel N., Korolevskaya L., Chereshnev V., Robinson J. (2016). Systemic inflammation and liver damage in HIV/hepatitis C virus coinfection. HIV Med..

[105] Aibibula W., Cox J., Hamelin A.-M., Moodie E.E., Anema A., Klein M.B. (2018). Association between depressive symptoms, CD4 count and HIV viral suppression among HIV-HCV co-infected people. AIDS Care.

[106] Fialho R., Pereira M., Harrison N., Rusted J., Whale R. (2017). Co-infection with HIV associated with reduced vulnerability to symptoms of depression during antiviral treatment for hepatitis C. Psychiatr. Res..

[107] Clifford D.B., Evans S.R., Yang Y., Gulick R.M. (2005). The neuropsychological and neurological impact of hepatitis C virus co-infection in HIV-infected subjects. Aids.

